# Analysis of Gene Expression Data Using BRB-Array Tools

**Published:** 2007-02-04

**Authors:** Richard Simon, Amy Lam, Ming-Chung Li, Michael Ngan, Supriya Menenzes, Yingdong Zhao

**Affiliations:** 1 Biometric Research Branch, National Cancer Institute, 9000 Rockville Pike, Bethesda MD; 2 Emmes Corporation, Rockville MD; 3 SRA International, Rockville MD

**Keywords:** Bioinformatics, microarrays, gene expression, biostatistics

## Abstract

BRB-ArrayTools is an integrated software system for the comprehensive analysis of DNA microarray experiments. It was developed by professional biostatisticians experienced in the design and analysis of DNA microarray studies and incorporates methods developed by leading statistical laboratories. The software is designed for use by biomedical scientists who wish to have access to state-of-the-art statistical methods for the analysis of gene expression data and to receive training in the statistical analysis of high dimensional data. The software provides the most extensive set of tools available for predictive classifier development and complete cross-validation. It offers extensive links to genomic websites for gene annotation and analysis tools for pathway analysis. An archive of over 100 datasets of published microarray data with associated clinical data is provided and BRB-ArrayTools automatically imports data from the Gene Expression Omnibus public archive at the National Center for Biotechnology Information.

## Introduction

The use of gene expression profiling has increased dramatically but serious problems in the analysis of such data in publications are prevalent (Dupuy and Simon, 2007; Michiels et al. 2005). Valid analysis of DNA microarray experiments requires substantial statistical knowledge but statisticians with expertise in microarray methods are in short supply and not available to many laboratories.

BRB-ArrayTools was developed in an attempt to broadly share the knowledge gained by biostatisticians of the Biometric Research Branch of the National Cancer Institute in a decade of involvement in collaborative microarray investigations and in the development of statistical methodology for microarray data. The primary objectives of BRB-ArrayTools are: (1) To provide scientists with software that guides them to utilize valid and powerful methods appropriate for their experimental objectives without requiring them to learn a programming language. (2) To encapsulate into software the experience of professional statisticians who read and critically evaluate the extensive published literature of new analytic and computational methods. (3) To facilitate education of scientists in statistical methods for the analysis of DNA microarray data.

The next section describes the architecture of BRB-ArrayTools. The following sections provide an overview of DNA microarray analysis and some of the analysis tools included in the system. The software also includes tutorials and microarray datasets. BRB-ArrayTools is available without charge for non-commercial applications at http://linus.nci.nih.gov/BRB-ArrayTools.html. More detailed information on the use of the software is contained in the User’s Guide (Simon and Lam, 2006) and more details on the statistical methods implemented in the software are contained in Simon et al. (Simon et al. 2003a).

## Architecture

**Figure 1.**
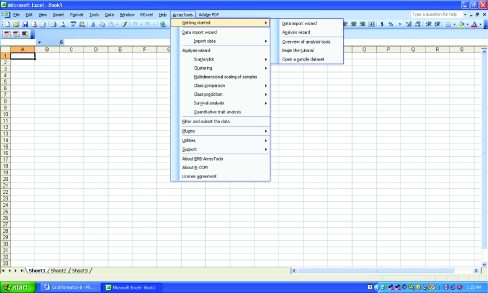
Screen shot of BRB-ArrayTools pull-down menu in Microsoft Excel.

The BRB-ArrayTools installer loads the package as an add-in to Microsoft Excel under the Microsoft Windows family of operating systems. The installation creates an ArrayTools menu on the Excel menu bar and all user interactions with BRB-ArrayTools are through the menu and dialog boxes which are launched by the selection of menu items (Fig. 1). Most of the analysis tools provided in BRB-ArrayTools are much too computationally intensive to be performed by Excel functions. The computing for these tools is performed by programs launched by menu selections written in either the R statistical programming language, or by compiled programs. Output of the analysis tools is presented to the user on HTML files or as dynamic interactive graphical objects.

## Study Objectives and Analysis Strategies

BRB-ArrayTools attempts to teach users that an effective analysis strategy must be tailored to the specific objectives of the investigation (Simon et al. 2003a). Many objectives fall into the categories of Class Comparison, Class Prediction or Class Discovery.

With ***class comparison*** the objective is to identify the genes that are differentially expressed among groups of specimens collected from different types of tissues or under different experimental conditions. Users are instructed that cluster analysis methods are not appropriate for class comparison objectives. Supervised methods, which take into account information about which samples are from which phenotype classes, are more powerful for identifying differentially expressed genes. Users are also instructed that for the valid application of supervised methods, they need to distinguish technical replicates from biological replicates. Broad biological conclusions require study of samples that reflect the full range of biological variability (Simon and Dobbin, 2002). Supervised methods also permit proper statistical control of the number of false positive findings. BRB-ArrayTools provides powerful methods for controlling false discoveries (Korn et al. 2004, Tusher et al. 2001).

***Class prediction*** is similar to class discovery in that the classes are pre-defined independently of the expression data. With class prediction, however, the emphasis is on developing a multi-gene classifier that enables one to predict the class of a sample based on its expression profile. Class prediction is often relevant to biomedical studies. For example, one may have a set of expression profiles for tumors of patients before receiving a specified treatment. Some patients respond to treatment and some do not. With class prediction the emphasis is developing an accurate profile classifier that can be used to predict whether a future patient will respond to treatment and obtaining an unbiased estimate of the prediction accuracy that can be expected for it.

***Class discovery*** refers to problems where there is not a pre-defined taxonomy of the samples and one wishes to determine whether the expression profiles fall into two or more characteristic clusters. Exploratory cluster analysis methods are used for grouping the samples into subsets which are relatively homogeneous with regard to expression profile. Cluster analysis methods are also used to group the genes into subsets which are relatively homogeneous with regard to their co-variation across the samples studied.

## Importing Data

BRB-ArrayTools contains a data import wizard that enables the user to easily import the data files created by image analysis programs used for any single label or dual label platform. BRB-ArrayTools contains automatic importers for some popular platforms, such as the Affymetrix GeneChip system. Affymetrix expression data can be imported either as raw CEL files or as processed probe summaries created by the Affymetrix GCOS or MASS5 software. BRB-ArrayTools also contains automatic importers for a variety of commonly used platforms and for all gene expression data and associated clinical and pathological annotations in the Gene Expression Omnibus public archive maintained by the National Center for Biomedical Technology. The BRB-ArrayTools website also provides an archive of over one hundred publicly available human tumor microarray datasets with associated clinical data already converted to BRB-ArrayTools format.

In addition to importing the single or dual channel intensities for each gene on each array, BRB-ArrayTools imports an experiment descriptor worksheet that the user creates to describe the phenotype attributes associated with each of the samples included in the experiment. The imported experiment descriptor worksheet enables the user to direct the analysis tools. Each row corresponds to a sample that was hybridized to an array and each column corresponds to a phenotype descriptor. The user can add columns at any time during the analysis.

The user may also import gene descriptors corresponding to the probes on the arrays. For Affymetrix data, a probe set identifier is contained in the expression data files and the program automatically obtains a wide variety of other descriptors from the NetAffyx website provided by Affymetrix. For printed arrays, if the user provides any single identifier such as a clone identifier, a Unigene cluster identifier, a gene symbol, or a Genbank accession number, the program will automatically obtain a wide variety of other gene annotations from the Source database (Diehn et al. 2003). The program also automatically imports Gene Ontology terms for all the genes represented on the arrays used, Kegg metabolic pathway and Biocarta signaling pathway information.

## Normalization

BRB-ArrayTools performs a variety of preprocessing steps including computing probe-set expression summaries, normalization, filtering and calculating quality control indices. For Affymetrix data the robust model based probe-set expression summaries are implemented utilizing Bioconductor software (Gentleman and Carey, 2002). In this way, BRB-ArrayTools makes these advanced analysis facilities available to biomedical scientists who are not familiar with the R programming language required for direct use of Bioconductor functions. BRB-ArrayTools also provides intensity dependent non-parametric normalization (Yang et al. 2002).

## Finding Differentially Expressed or Prognostic Genes

DNA microarrays are commonly used for identifying the genes differentially expressed between two or more different tissue types or experimental conditions. The class comparison tool of BRB-ArrayTools provides powerful methods for finding differentially expressed genes while controlling either the number or proportion of false discoveries. The multivariate permutation tests used, described in detail in (Korn et al. 2004; Simon et al. 2003a), enable the user to specify, for example, that there should be 90% confidence that the resulting gene list contains no more than 10% false discoveries. This method is similar to the popular Statistical Analysis of Microarrays (SAM) method (Tusher et al. 2001) but provides greater probabilistic control of the false discovery rate. The individual gene test statistics are based on a hierarchical model that enables within-class variance information to be shared among genes in a manner that does not assume that all genes have the same variance (Wright and Simon, 2003). The multivariate permutation test is fully non-parametric and is much more powerful than standard univariate permutation tests, particularly when the number of biological replicates within classes is small. The method is more robust than parametric t or F tests used in the analysis of variance. The multivariate permutation test also takes advantage of the correlation structure of the genes. The SAM method (Tusher et al. 2001) is also available within BRB-ArrayTools, and is implemented as a compiled FORTRAN program that runs several times faster than the other versions of SAM.

The basic class comparison tool has several options including the ability to stratify the analysis by a potentially confounding variable and the ability to do paired analyses. BRB-ArrayTools contains several other tools for finding differentially expressed or prognostic genes in more complex settings. For example, the quantitative trait tool finds genes significantly correlated with a quantitative phenotype such as age. The survival tool finds genes significantly associated with right censored survival data. In all of these tools, the multivariate permutation approach is used to provide a confidence-specific control on the number or proportion of false discoveries. BRB-Array Tools also provide analysis of variance tools for time-course analysis, for settings with numerous phenotypic factors of interest, fixed effect models, mixed models with random effects, and models for analysis of complex dual label hybridization designs that do not use a common reference sample.

The output of each tool is a list of significant genes, with numerous annotations for the genes and links to websites containing additional information. Included in the annotations are Gene Ontology categories and an analysis of which categories are over-represented in the gene list relative to the prevalence of Gene Ontology categories on the array. Chromosome location and pathway analyses are also provided.

In addition to using Gene Ontology descriptors to annotate a gene list, BRB-ArrayTools provides a tool for directly evaluating differential expression of Gene Ontology categories, Kegg or Biocarta pathways, Broad Institute signatures or user specified gene lists. This gene set comparison analysis is similar to the gene set enrichment analysis described by Subramanian et al. (Subramanian et al. 2005). It reduces the number of comparisons, and thereby the multiple testing penalty, since inference is not made for individual genes. It also enables the discovery of differentially regulated pathways even where individual genes do not have large enough fold differences to be individually identified (Mootha et al. 2003).

## Class Prediction

Predictive modeling is an area in which there is considerable confusion in the microarray literature (Simon et al. 2003b). Most statistical classification methods were not developed for applications in which the number of candidate predictors (p) is orders of magnitude greater than the number of cases (n), and many standard statistical approaches do not perform well in this setting. For p ≫ n microarray settings, the usual practice of using the same data to develop and test a predictive model is almost guaranteed to give misleading results. When p ≫ n, even with completely random data it is always possible to identify features and develop a model that perfectly fits the data. But such a model will be completely useless for prediction with independent data. Consequently, with microarray data it is essential to evaluate a classifier model using data that was not used to select the genes or fit the model.

There are several prevalent misconceptions about the development and validation of classification models for microarray data. One misconception is that complex classifiers are better than simpler classifiers. Most comparative studies have shown that simple classifiers generally perform at least as well as more complex methods for p ≫ n settings (Ben-Dor et al. 2000; Dudoit et al. 2002). There is also a misconception that it is valid to select genes using all of the data and then to cross-validate the determination of the model parameters for the reduced set of genes. This partial cross-validation is widely used (Ntzani and Ioannidis, 2003) but has been shown to be highly biased and misleading (Ambroise and McLachlan, 2002; Simon et al. 2003b).

BRB-ArrayTools provides automatic complete cross-validation of the model development process. The models provided include diagonal linear discriminant analysis (Dudoit and Fridlyand, 2003), compound covariate predictor (Radmacher et al. 2002), nearest neighbor classification (Ripley, 1996), nearest centroid classification, shrunken centroid classification (Tibshirani et al. 2002), support vector machines (Ramaswamy et al. 2001), random forest classification (Breiman, 2001), top scoring pairs classification (Geman et al. 2004) and the Bayesian compound covariate predictor (Wright and Simon, 2003).

The complete cross-validation methods provided for estimation of the generalization error rate include leave-one-out cross-validation (Lachenbruch and Mickey, 1968), repeated k-fold cross-validation (Burman, 1989), split-sample validation and 0.632+ bootstrap validation (Efron and Tibshirani, 1997). Gene selection is repeated in all re-sampling and bootstrap validation procedures.

BRB-ArrayTools provides a permutation significance level for the cross-validated error estimate (Radmacher et al. 2002). For each random permutation of class labels, the entire cross-validation procedure is repeated. The proportion of the random permutations that provide a cross-validated error rate no greater than that for the true set of class labels is the permutation significance level.

## Survival Risk Group Prediction

Many clinical studies address the problem of predicting prognosis by artificially dividing survival or progression-free survival data into discrete classes and then using class prediction methods. This entails loss of information and is not necessary. BRB-ArrayTools includes a tool for directly predicting survival risk group based on expression data, for obtaining cross-validated Kaplan-Meier survival curves and for computing a permutation significance level for the separation among the cross-validated Kaplan-Meier survival curves. This tool is based on proportional hazards modeling in which the expression data is represented by the first several principal components of the genes whose expression is correlated with survival outcome, i.e. supervised principal components (Bair and Tibshirani, 2004). The analysis tool also provides for a comparison of the ability of expression data to predict survival risk to that of standard clinical and pathological prognostic factors and also provides the user with the option of developing survival risk predictors that utilize both gene expression data and clinical or pathological prognostic factors.

## Cluster Analysis

Cluster analysis is a useful descriptive tool although it is often applied in settings where supervised methods are more appropriate. BRB-ArrayTools provides the user with a variety of cluster analysis tools and also incorporates the popular Cluster and TreeView tools (Eisen et al. 1998). BRB-ArrayTools includes methods for evaluating the robustness and statistical significance of clusters of samples, important tools that are rarely provided in other software (McShane et al. 2002).

## Graphical Displays

**Figure 2.**
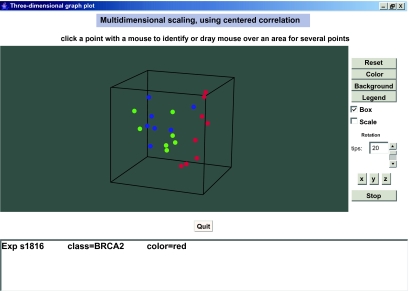
Screen shot of rotating 3-dimensional multidimensional scaling plot from BRB-ArrayTools. Each point represents a sample. The three axes are the first three principal components of the genes, representing the three dimensions of the expression profile of the samples having maximum variability. Samples are color coded by a characteristic selected by the user. In the case shown, the characteristic is presence of BRCA1 germline mutation (green), presence of BRCA2 germline mutation (red), or neither (blue). The direction and speed of rotation is controlled by the user. Points can be identified by clicking with mouse. The entire graphical display, including operating controls, can be automatically exported to Microsoft Powerpoint.

BRB-ArrayTools contains a wide range of useful graphical displays for use in quality assurance and analysis. For example, Figure 2 shows a snapshot of the rotating 3-dimensional multidimensional scaling analysis available in BRB-ArrayTools. In this display each sample is represented by a point. The axes are the linear combinations of the genes which best preserve the inter-sample distances. In most cases, the axes correspond to the first three principal components of the genes. The points are color-coded using any column of the experiment descriptor file specified by the user. The plot is rotating and the user can control the axes and speed of rotation. Rotation is essential to observe the three dimensional structure. The entire plot can be automatically exported to PowerPoint by a command provided in BRB-ArrayTools. The rotating plot, including all controls is exported and can be controlled during a PowerPoint presentation.

## Extensibility

BRB-ArrayTools contains a plug-in feature that enables statistical users to extend the software using their own functions written in the R statistical language. The plug-in facility also provides the plug-in developer with a wizard for creating a graphical user interface for interacting with the BRB-ArrayTools user. Hence the developer who knows the R statistical language can create a professional user interface that will enable his/her function to be distributed and used by the larger community of BRB-ArrayTools investigators.

## Discussion

BRB-ArrayTools represents a novel experiment in enabling scientists to equip themselves to take advantage of the revolutionary changes taking place in biology. There are currently over 5000 registered users in over 60 countries. Several hundred publications have cited BRB-ArrayTools as being used for their data analysis. The program is actively being extended to provide additional tools for gene expression analysis. Our judgments of what to include in BRB-ArrayTools are heavily influenced by our involvement in experimental and methodologic microarray research, in the design and analysis of a variety of large and small experiments, and interactions with our users. Being very much involved in the development of new statistical methods for this type of research forces us to attempt to stay current with the extensive literature in the statistical, bioinformatics and machine learning literature and to critically evaluate which methods represent important advances for the field and should be included in our software. As important as the inclusion of new tools, however, is the effectiveness of the software for helping biomedical scientists educate themselves in the use of advanced statistical and bioinformatic methods to prepare themselves for the revolution that is taking place in biology.
